# Self-palpation for detection of paroxysmal atrial fibrillation: Much noise with little signal

**DOI:** 10.1371/journal.pmed.1003098

**Published:** 2020-03-31

**Authors:** Kazem Rahimi, Paulus Kirchhof

**Affiliations:** 1 Deep Medicine, Big Data Insitute, Nuffield Department of Population Health, University of Oxford, Oxford, United Kingdom; 2 NIHR Oxford Biomedical Research Centre, Oxford University Hospitals NHS Foundation Trust, Oxford, United Kingdom; 3 Institute of Cardiovascular Sciences, University of Birmingham, Birmingham, United Kingdom; 4 Department of Cardiology, University Heart and Vascular Center Hamburg, Hamburg, Germany; 5 SWBH and UHB NHS Trusts, Birmingham, United Kingdom; 6 AFNET, Münster, Germany

## Abstract

Kazem Rahimi and Paulus Kirchhof discuss the latest work from Faris Ghazal et al and how it may impact the way we currently detect Atrial Fibrillation in the general population.

Patients with atrial fibrillation (AF) are at substantially increased risk of various adverse cardiovascular events, such as stroke, myocardial infarction, heart failure, and vascular dementia [1]. Given that such risks can be partially mitigated through preventive measures, it is important to understand how to better identify people affected by AF and how better identification might change treatment decisions or outcomes.

The cardinal feature of AF is the presence of an irregular pulse. Advocating for pulse palpation might, then, seem a simple and ubiquitously available solution for detecting undiagnosed AF. Indeed, previous research has shown that such a simple screening method, when adopted by trained health professionals, is as good as systematic screening with the use of ECG [2]. Hence, systematic pulse palpation is recommended during routine clinical encounters of at-risk patients [3,4]. However, although such screening will detect chronic forms of AF when patients are seen by health professionals, it neither detects intermittent, paroxysmal AF nor facilitates the diagnosis of AF in populations not assessed by health professionals. These “holes in the current screening net” might be plugged by regular self-pulse palpations by laypersons.

In this context, Faris Ghazal and colleagues compared self-pulse palpation to simultaneously recorded ECGs in an important study reported in *PLOS Medicine* [5]. They recruited around 1,000 individuals aged 65 years and over from 4 primary care centres in Sweden. All participants were trained on how to monitor irregular pulse rhythms 3 times a day over a 2-week period. Importantly, they were also given a handheld ECG device and were advised to record their heart rhythm simultaneously with self-palpation. This resulted in 53,782 simultaneous pulse palpations and ECG recordings (51 recordings per individual). Intermittent ECG recordings were confirmed to be effective in detecting 27 patients with AF (2.7%, 95% CI 1.8–3.9). Only a small minority of recordings (42 patients) required verification with a heart rhythm patch that was worn over 5 days (with AF being verified in just 4 of these patients, i.e., 10%). All but 2 individuals had paroxysmal AF, illustrating the power of patient-operated ECG devices.

But how well did self-palpation perform? In short, not very well. Only 15 of the 27 individuals with newly diagnosed AF reported at least 1 episode of irregular pulse during the screening period. This translates to a sensitivity of 56% (95% CI 35%–75%) for diagnosis of AF by self-palpation at the individual level or 25% (95% CI 20%–30%) at the level of episodes of self-palpation. More importantly, the positive predictive value of self-palpation was disappointingly low (7%, 95% CI 5%–10%). This means that for each correctly detected irregular pulse, there were about 13 false alerts. This relationship did not change when the cumulative detection rate for each participant was considered: for each correctly diagnosed participant with AF, there were 12 participants who felt an abnormal rhythm on at least 1 occasion that could not be verified in simultaneous ECG recordings. This stable error rate suggests that the positive predictive value of self-palpation could not be increased by simply changing the duration or frequency of the screening period. Consequently, the adoption of lay self-palpation would likely lead to a substantially increased downstream demand for verification of false alarms, which will render an apparently low-cost strategy unsuitable for resource-constrained health systems. Furthermore, in routine clinical practice, the training of patients in self-palpation might not be of comparable quality as in Ghazal and colleagues’ study, and given that verification of heart rhythm is likely to be asynchronous to self-palpation episodes, a subsequent lack of clinical confirmation of a paroxysmal abnormal rhythm might create substantial anxiety among patients. Thus, this study provides clear evidence against a policy of routine self-palpation for detection of paroxysmal AF.

On the other hand, this study confirmed prior reports that patient-operated intermittent ECG recordings with handheld devices are effective for diagnosing paroxysmal AF [6,7]. Such devices may soon be replaced by emerging consumer applications, implantable devices, or various predictive algorithms that will enable detection of ever more subtle episodes of AF at lower unit costs [8–10]. These could facilitate the detection of the rare paroxysms of AF presently found in 15%–30% of elderly patients when continuous, long-term monitoring is applied [11,12].

However, the question of the consequence of more accurate and comprehensive AF detection and how to manage patients once atrial arrhythmias have been correctly identified is more complicated than might be assumed from the scope of this study. To assess these implications, we not only need to have confidence in the performance of the diagnostic tool but also need information on whether the treatment of subclinical, screening-detected forms of AF is beneficial for patients. Even putting the cost, inconvenience, and potential harm of any treatment aside, the extrapolation of benefits of treatments established among those identified through clinical encounters to those diagnosed through screening is prone to error, in part because of methodological challenges such as lead time and overdiagnosis bias. Such challenges are now well known to the oncology community, in which efforts to diagnose slow-progressing cancers early have not improved outcomes materially and, in some instances, caused avoidable harm [13]. In this vein, the conventional wisdom, which considers AF as a binary phenomenon that should be managed in the same way irrespective of how it was diagnosed and in whom, seems no longer tenable [12,14,15]. Fortunately, several controlled trials evaluating patients with potentially milder forms of AF are underway, for instance, testing anticoagulation therapy in patients with rare episodes of AF detected by implanted device [16,17] or in patients with AF detected by lay screening [18]. Such studies will likely provide reliable information on the impact of wider screening strategies for AF detection and treatment.

However, until such evidence becomes available, those who seek to detect even shorter spells of irregular heart rhythm will have to face the ambiguity of the meaning of the diagnosis and the uncertainty of its consequences. Notwithstanding, patients and consumers should be advised that we do have greater certainty on how to measure and modify cardiovascular risk despite the uncertainties surrounding the detection of short spells of AF outside clinical settings.
